# Severe Anemia and Helicobacter Pylori Infection in school age Children; A case reports

**Published:** 2016-03-15

**Authors:** Sh Gheibi, M Noroozi, S Hejazi, M Karamyyar, H Farrokh-Eslamlou

**Affiliations:** 1 **Maternal and Childhood Obesity Research Center, Urmia University of Medical Sciences, Urmia, Iran.**; 2 **Maternal and Childhood Obesity Research Center, Urmia University of Medical Sciences, Urmia, Iran.**; 3 **Department of Pediatric. Urmia University of Medical Sciences, Urmia, Iran.**; 4 **Maternal and Childhood Obesity Research Center, Urmia University of Medical Sciences, Urmia, Iran.**; 5 **UrmiaReproductive Health Research Center, School of Public Health, Urmia University of Medical Sciences, Kashani Street, Urmia, Iran.**

**Keywords:** Helicobacter pylor, Iron deficiency, Severe Iron deficiency anemia

## Abstract

**Background:**

Iron-deficiency anemia is a widespread public health problem with major consequences for human health especially, children. However, in a fraction of patients an underlying cause is never found during routine investigation. Recent studies have suggested an association between Helicobacter pylori (H. Pylori) infection and iron-deficiency anemia.

**Case presentation:**

Here is reported four school aged children (two male, two female) with refractory severe iron-deficiency anemia associated H. Pylori gastritis. Mean age of the patients was 13.62 years old and they were admitted with chief complaints of abdominal, chest pain weakness, headache and respiratory distress.

Mean hemoglobin level in patients was 6.2 g/dl with persistence to iron therapy. After the diagnosis and therapy of H. pylori infection, clinical complaints, hemoglobin level and iron profiles were being normal and they gained weight.

**Conclusion:**

This study suggests screening of H. pylori infection and appropriate treatment in any case of refractory moderate to severe iron-deficiency anemia, especially with clinical manifestations of gastrointestinal tract in children.

## Introduction

The global prevalence of Helicobacter pylori (H. Pylori) infection is more than 50% (1) so it is the most common infection through the world (2). The prevalence may vary significantly in relation to geography, ethnicity, age, and socioeconomic factors (3-5). H. Pylori infection is very prevalent in developing countries and it is markedly more revalent at younger ages and those of lower socioeconomic status (6). Colonization of H. Pylori is usually asymptomatic, but approximately 20% of the infected population evolves into a chronic gastritis and peptic ulcer (7). H. Pylori infection in children has mostly been associated with recurrent abdominal pain, gastric dyspepsia, or duodenal ulcer (8). Uncommon clinical features such as protein-losing enteropathy (9) and malabsorption (10) have also been reported. Although without a clear explanation, other extra-digestive tract conditions such as iron deficiency (ID) or iron deficiency anemia (IDA) have been recently related to the H. Pylori infection (11). Some studies have suggested that specific strains of H. pylori have enhanced iron ion uptake ability and are associated with IDA (12). The anemia was refractory to iron therapy and reversed only after bacteria eradication (13), thus suggesting possible interference of H. pylori in iron metabolism (14, 15). In order to understand the possible pathogenic role of H. pylori in development of the anemia, extensive investigations have been undertaken (16). A review of the English-written literature revealed only few reports of severe anemia associated with H. Pylori infection in children and adolescents (17-21). Here is presented a series of four consecutive similar cases of severe iron deficiency anemia associated with H. Pylori infection that expands upon the clinical and endoscopic features of H. Pylori in children.

## Case Report

**Case 1:** A 13 years old school girl who was referred to the hospital with clinical symptoms of chest and abdominal pain and a refractory iron deficiency anemia since two months before admission. Other medical histories and also menses bleeding were normal. On the Physical Examination (P/E), she was 28kg, pale but with a good general appearance. She has normal breathing sounds, but cardiac auscultation appeared mildly tachycardia (HR=98/min). Abdomen was soft except for epigastric tenderness on palpation. Her Chest X. Ray (CXR) and echocardiography were normal. Laboratory analysis showed a hemoglobin (Hb) level of 6.2 g/dl, Mean Corpuscular Volume (MCV) level of 58.6 fL, a serum iron (SI) level of 29 μg/dl, a Total Iron Binding Capacity (TIBC) of 416 μg/dl, a ferritin level of 3 ng/mL, and a reticulocyte count of 0.3%. Serology study revealed an anti H. Pylori (AHP) IgG level of 104 U/ml (Nl <20), and an anti H. Pylori IgM level of 48 U/ml (Nl <40). After transfusion of 280 ml isogroup and cross matched packed cell an upper gastrointestinal endoscopy was performed that showed antral gastritis with positive Rapid Urease Test (RUT). Pathologic examination reported chronic gastritis with H. Pylori. Following these findings, the patient received medicaments including clarithromycin (20mg/kg), amoxicillin (50mg/kg) and omeprazole (1mg/kg) for two weeks followed by omeprazole (20mg/daily) alone for another two months. After this therapy she became symptom free. During the six months of follow- up, she gained weight up to 34 kg, the Hb and MCV values raised to 13.3 g/dL and 85 fL, respectively.

**Case 2:** A 14 years old school boy has been presented with weakness and headache since 6 months ago and a refractory iron deficiency anemia for 2 months. He was admitted to the hospital due to transient hyperglycemia (a blood sugar of 196 mg/dl) and has been investigated to find out the etiology of anemia. In general appearance, he was cachectic and pale. On physical examination, pulse rate (PR) and respiratory rate (RR) and weight were 94/min, 26/min and 49 kg, respectively. He had epigastric tenderness. His admission Hb, MCV, SI, TIBC and ferritin were 5.9 g/dl, 63 fL, 18 μg/dl, 447 μg/dl and 10 ng/mL, respectively. AHP IgG level was 64 U/ml (Nl <20), and AHP IgM level was 84 U/ml (Nl <40). 

Esogastroduodenoscopy (EGD) was done following the transfusion of 450 ml of packed cell. A Gastro-Esophageal Reflux Disease (GERD) and gastritis were reported in EGD. Rapid urease test was positive too. Histopathology of given samples reported a chronic gastritis with focal activity and H. pylori presence. He received clarithromycin, amoxicillin, and omeprazole for two weeks followed with omeprazole alone for subsequent two months. After treatment just the initial symptoms of patient were relieved and weight was increased to 55 kg.

**Case 3:** A 13.5 years old girl was referred with the chief complaint of respiratory distress during activity plus an abdominal pain since 3 months before admission. A normal vital sign, paleness and epigastric tenderness were found in the physical examination. Her weight was 51 kg. Her CXR was normal. Laboratory tests revealed an Hb=6.5 g/dL, MCV=61 fL, AHP IgG=33 U/ml and AHP IgM=92 U/ml. Following transfusion of 450 ml of packed cell, EGD was done. She developed gastritis and positive rapid urease test. Histopathology analysis reported a chronic gastritis with intestinal metaplasia and H. pylori infection. After receiving a triple therapy similar to other patients, her initial symptoms were relieved and her weight gets to 54kg. After six months of treatment, her hemoglobin level and other laboratory findings became normal.

**Case 4:** A 14 years old boy was admitted to the hospital because of vague abdominal pain and fatigue for one year. There was a history of receiving oral ferrous sulfate and multivitamins for three months about six months before admission. His physical examination revealed paleness and epigastric tenderness. Laboratory investigations showed a hemoglobin level of 6.2 g/dl, a MCV level of 63 fl, a serum iron level of 23μg/dl, a TIBC level of 520 μg/dl, an AHP IgG level of 72 U/ml and an AHP IgM level of 46 U/ml. Upper EGD showed GERD, gastritis, duodenal ulcer and positive RUT. Histopathology findings showed a chronic gastritis with H. Pylori infection. He received triple H. Pylori eradication therapy for 2 weeks followed by omeprazole for three months. He became symptom free and his weight increased about five kg to reach 43 kg. His hematologic tests and iron profiles became to normal range after 6 months. **Table I **summarized some characteristics of the four cases.

**Table II:** *Basic characteristics of patients with severe anemia and H. Pylori infection*

patient	Gender	Age (year)	Hemoglobin level at admission (g/dl)	**S**ymptom	Weight gain (kg)
Case1	female	13	6.2	Chest and abdominal pain	6
Case2	male	14	5.9	Weakness and headache	6
Case3	female	13/5	6.5	Respiratory distress	3
Case4	male	14	6.2	Abdominal pain and Paleness	5

## Discussion

H. pylori infection and iron deficiency anemia are prevalent in disadvantaged populations worldwide (22). Numerous studies have suggested a link between iron-deficiency anemia (IDA) and H. pylori infection (12,13,20,23,24). Researcher believed that H. pylori infection may play a role in iron-deficiency anemia (25). H. pylori gastritis is a frequent but underdiagnosed cause of refractory anemia in teenagers (13). For the first time, Bruel et al, (18) in 1993 reported an 11 years old patient with severe anemia (hemoglobin level of 5.6 g/dl) due to upper digestive tract bleeding, hemorrhagic inflammation of the duodenum and antritis with positive urea test and diagnosis of H. pylori infection on the smears. Laboratory tests showed low serum iron and ferritin levels. Nowicki et al (17), in 2001 reported an uncommon presentation of H. pylori infection in a child with severe iron deficiency anemia and asymptomatic nodular gastro-duodenitis. Also, Ashorn et al in 2001 reported eight school-age children with refractory iron deficiency anemia while their gastroscopy revealed H. pylori infection and a quadruple eradication therapy for H. pylori was prescribed. After treatment, hemoglobin level was corrected. In acceptance to us, they suggested that H. pylori might have a role in causing iron deficiency anemia in school-age children (26). Then Cardamone et al (21) in 2008 reported three children with symptomatic refractory iron-deficiency anemia without an obvious clinical etiology. All children underwent a diagnostic endoscopy and were found to have H. pylori gastritis. They concluded, H. pylori infection was an importance cause of refractory iron-deficiency anemia in adolescents.This study was reported a series of four school children with refractory iron deficiency anemia which hemoglobin level and iron indexes as well as their clinical symptoms were improved following anti-H, Pylori therapy. Present results confirmed the findings of Konno et al in 2000 in Japan (20), which reported an IDA recurrence after discontinuation of the iron therapy in six patients. They found a marked antral nodularity without evidence of bleeding lesions during EGD in all the patients. Positive rapid urease test and chronic active gastritis with presence of H. pylori confirmed the diagnosis of H. Pylori infection. Hematologic profile and iron status were improved after triple therapy consisting of lansoprazole, clarithromycin, and metronidazole and there was no evidence of IDA between 27 and 50 months follow up (23). Present case series reveals that severe and refractory iron deficiency anemia can precipitate with H. Pylori infection in children. These patients did not respond to treatment with iron products. Several possible mechanisms for the association between anemia and H. Pylori infection have been suggested (27-29). Intermittent bleeding is one possibility that may be present (14), although in these four patients no bleeding lesions in the duodenum and stomach or occult bleeding was detected in stool specimens during the follow-up periods. Another possible reason could be that H. Pylori may have an iron-acquisition mechanism in vivo, forming a parasitic relationship to compete with the host for iron. It is well known that microorganisms need the hosts iron to grow (12). Additional clinical observations and studies are needed on the bacterium strains to explain why IDA could be only a rare consequence of such a frequent infection.

## Conclusion

This study suggests screening of H. pylori infection and appropriate treatment in any case of refractory moderate to severe iron-deficiency anemia, especially with clinical manifestations of gastrointestinal tract in children.

## Conflict of interests

The authors have no conflicts of interest to disclose.
